# Supplementary material for: Left Ventricular Hypertrabeculation (LVHT) in Athletes: A Negligible Finding?
Source: Medicina (Kaunas). 2024 Dec 28;61(1):32. doi: 10.3390/medicina61010032 (PMC11767094; doi:10.3390/medicina61010032)
Supplement: Supplementary file 1 [file medicina-61-00032-s001.zip › Legend.pdf]

Supplemental Videos (Legend)

Video S1. Transthoracic echocardiography: 4 chamber heart view.

Video S2. Transthoracic echocardiography: modified 4 chamber heart view with zoomed both ventricles.

Video S3. Transthoracic echocardiography: short axis at midventricular level.

Video S4. Coronary artery computed tomography angiography representing normal coronary arteries.

Video S5. Cardiac magnetic resonance cine (SSFP) sequence: 4 chamber heart view representing left ventricular and right ventricular hypertrabecularisation.

Video S6. Cardiac magnetic resonance cine (SSFP) sequence: short axis view at midventricular to apical level representing increase in left ventricular noncompacted to compacted layer ratio in lateral and inferior walls.

Video S7. Cardiac magnetic resonance cine (SSFP) sequence: short axis view at apical level representing increase in left ventricular noncompacted to compacted layer ratio in all walls except septum.

Video S8. Cardiac magnetic resonance cine (SSFP) sequence: 4 chamber heart view representing decrease in left ventricular end-diastolic diameter and noncompacted to compacted layer ratio.

Video S8. Cardiac magnetic resonance cine (SSFP) sequence: short axis view at midventricular to apical level representing decrease in left ventricular end-diastolic diameter and noncompacted to compacted layer ratio.

Video S10. Cardiac magnetic resonance cine (SSFP) sequence: short axis view at apical level representing decrease in left ventricular end-diastolic diameter and noncompacted to compacted layer ratio.
