# Supplementary figures and images for: Left Ventricular Hypertrabeculation (LVHT) in Athletes: A Negligible Finding?
Source: Medicina (Kaunas). 2024 Dec 28;61(1):32. doi: 10.3390/medicina61010032 (PMC11767094; doi:10.3390/medicina61010032)

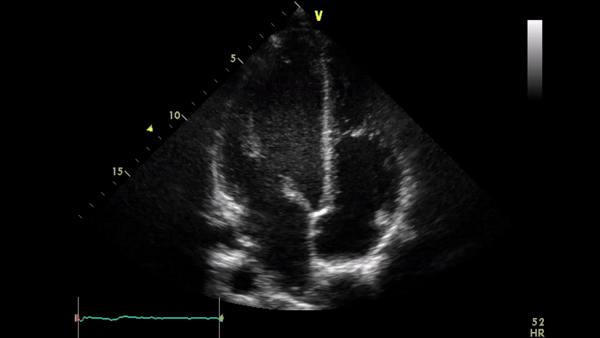

Supplement: Supplementary file 1 [file medicina-61-00032-s001.zip › Supplemental Video S1-GIF.gif]

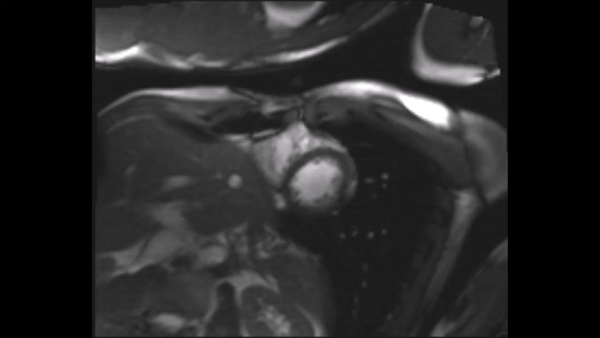

Supplement: Supplementary file 1 [file medicina-61-00032-s001.zip › Supplemental Video S10-ezgif.com-video-to-gif-converter.gif]

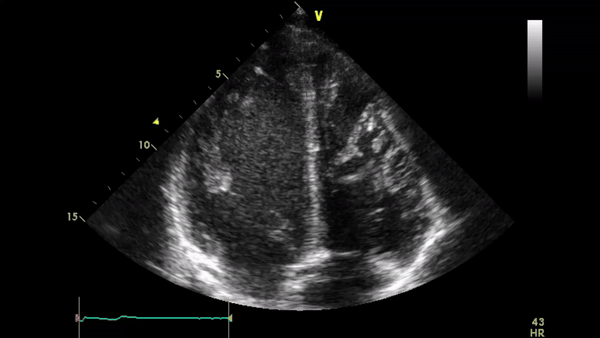

Supplement: Supplementary file 1 [file medicina-61-00032-s001.zip › Supplemental Video S2-GIF.gif]

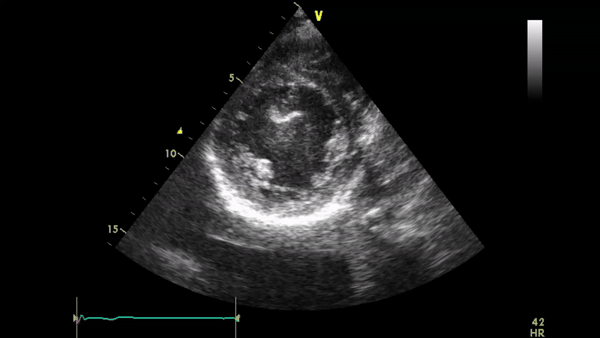

Supplement: Supplementary file 1 [file medicina-61-00032-s001.zip › Supplemental Video S3-GIF.gif]

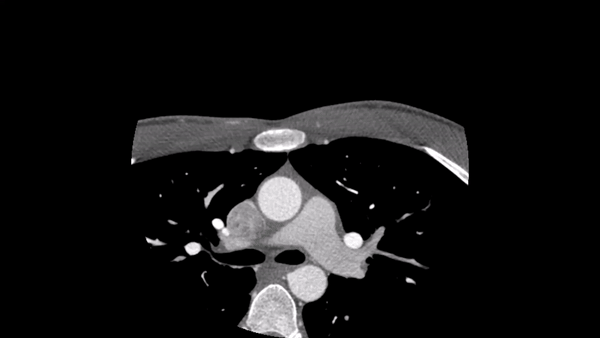

Supplement: Supplementary file 1 [file medicina-61-00032-s001.zip › Supplemental Video S4-GIF.gif]

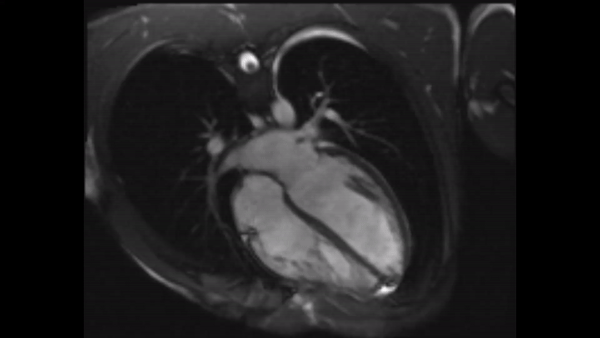

Supplement: Supplementary file 1 [file medicina-61-00032-s001.zip › Supplemental Video S5-ezgif.com-video-to-gif-converter.gif]

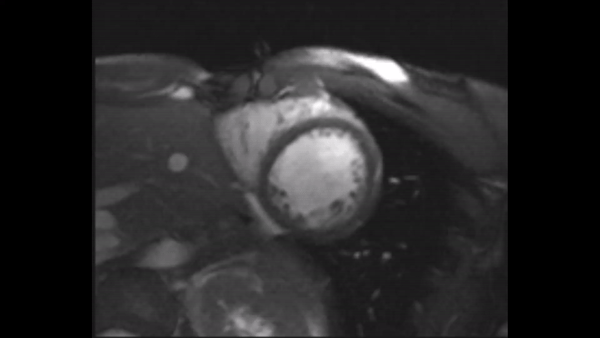

Supplement: Supplementary file 1 [file medicina-61-00032-s001.zip › Supplemental Video S6-ezgif.com-video-to-gif-converter.gif]

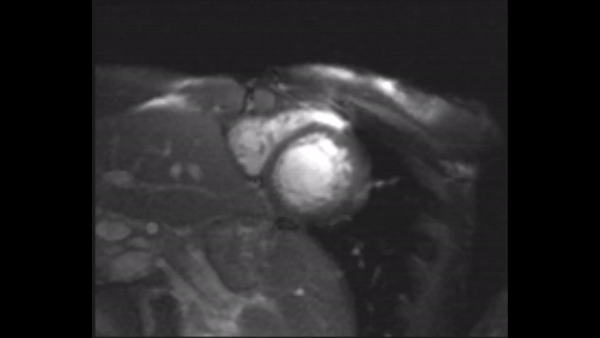

Supplement: Supplementary file 1 [file medicina-61-00032-s001.zip › Supplemental Video S7-ezgif.com-video-to-gif-converter.gif]

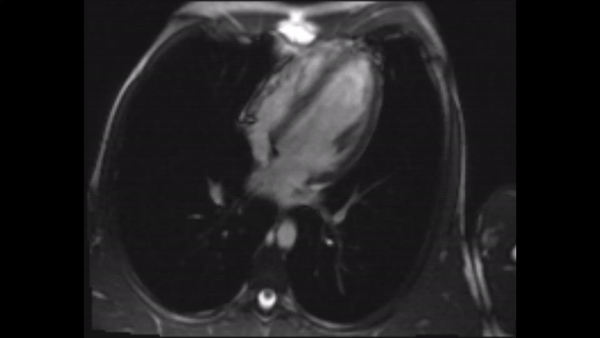

Supplement: Supplementary file 1 [file medicina-61-00032-s001.zip › Supplemental Video S8-ezgif.com-video-to-gif-converter.gif]

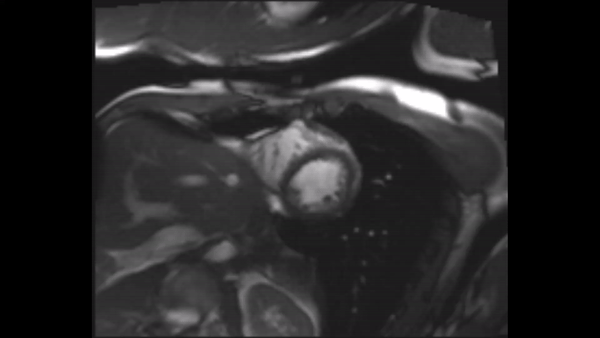

Supplement: Supplementary file 1 [file medicina-61-00032-s001.zip › Supplemental Video S9-ezgif.com-video-to-gif-converter.gif]
